# A novel strategy for site selective spin-labeling to investigate bioactive entities by DNP and EPR spectroscopy

**DOI:** 10.1038/s41598-021-92975-6

**Published:** 2021-07-01

**Authors:** Kevin Herr, Max Fleckenstein, Martin Brodrecht, Mark V. Höfler, Henrike Heise, Fabien Aussenac, Torsten Gutmann, Michael Reggelin, Gerd Buntkowsky

**Affiliations:** 1 Institute of Physical Chemistry, Technical University Darmstadt, Alarich-Weiss-Straße 8, 64287 Darmstadt, Germany; 2 Institute of Organic Chemistry, Technical University Darmstadt, Alarich-Weiss-Straße 4, 64287 Darmstadt, Germany; 3 Structural Biochemistry (ICS-6), Institute of Complex Systems, Forschungszentrum Jülich, 52425 Jülich, Germany; 4 Institut für Physikalische Biologie, Heinrich-Heine-Universität Düsseldorf, 40225 Düsseldorf, Germany; 5 Bruker France SAS, 34 rue de l’industrie, 67160 Wissembourg, France

**Keywords:** Biophysical chemistry, Chemical physics, Synthetic chemistry methodology

## Abstract

A novel specific spin-labeling strategy for bioactive molecules is presented for eptifibatide (integrilin) an antiplatelet aggregation inhibitor, which derives from the venom of certain rattlesnakes. By specifically labeling the disulfide bridge this molecule becomes accessible for analytical techniques such as Electron Paramagnetic Resonance (EPR) and solid state Dynamic Nuclear Polarization (DNP). The necessary spin-label was synthesized and inserted into the disulfide bridge of eptifibatide via reductive followed by insertion by a double Michael addition under physiological conditions. This procedure is universally applicable for disulfide containing biomolecules and is expected to preserve their tertiary structure with minimal change due to the small size of the label and restoring of the previous disulfide connection. HPLC and MS analysis show the successful introduction of the spin label and EPR spectroscopy confirms its activity. DNP-enhanced solid state NMR experiments show signal enhancement factors of up to 19 in ^13^C CP MAS experiments which corresponds to time saving factors of up to 361. This clearly shows the high potential of our new spin labeling strategy for the introduction of site selective radical spin labels into biomolecules and biosolids without compromising its conformational integrity for structural investigations employing solid-state DNP or advanced EPR techniques.

## Introduction

In the medical context the field of drug delivery with its selective application of bioactive substances is becoming more and more attractive^1–3^. Targeted drug delivery is strongly connected with the selective accumulation or release of a drug at one or more desired binding sites^4^. However, monitoring the pharmacokinetics of drugs appears to be the major challenge of targeted drug delivery^5^. Labeling of drug molecules facilitates this monitoring drastically.

Chemical labels establish covalent and stable bonds with reactive residues and specific amino acids of biomolecules. Due to the binding strategy, they are robust, easy to handle and offer maximum efficiency with a broad variety of potential target sites for covalent coupling^6–11^.

In particular peptide based agents receive more and more attention as bioactive molecules for diagnostic imaging^12^. They have several advantages including facile synthesis, small sizes, tuneable targeting properties, lower immunogenicity and cytotoxicity^13^. They are employed in imaging methods such as gamma camera scintigraphy^14^, MRI (magnetic resonance imaging)^15^ and PET (positron emission tomography)^16^. In this context hyperpolarized molecules are interesting to boost the sensitivity of NMR (nuclear magnetic resonance) and MRI. The technique of dynamic nuclear polarization (DNP) transfers the three order of magnitude higher spin polarization of unpaired electrons into nuclear spin polarization. This allows a significant enhancement of the sensitivity in NMR experiments^17–19^. Dissolution DNP NMR combined with the HyperSense polarizer has further opened up the field of medical applications for DNP imaging employing silica bound radicals in order to facilitate an easy separation of the potentially harmful radicals from the hyperpolarized substrate^20–25^. Here it would be advantageous to employ biocompatible radicals, which do not need to be separated before in-vivo application of the hyperpolarized substrates. A particularly attractive approach is the usage of bioactive radicals, which combine the physiological function, e.g., inhibition of an enzyme, with attached spin labels. In order to make this approach successful it is necessary to affect the molecular structure and reactivity of the inhibitor to the smallest extent. Nowadays, most DNP enhanced NMR experiments are performed with biradical systems, because of their higher hyperpolarization efficacy. However, for this bioactive spin-labels it seems advantageous to sacrifice a part of the hyperpolarization and to employ smaller, sterically less demanding monoradicals such as TEMPO ((2,2,6,6-tetramethylpiperidin-1-yl)oxyl) or PROXYL ((2,2,5,5-tetramethylpyrrolidin-1-yl)oxyl), which have less influence on the structure of biomolecules^26–32^.

The introduction of these paramagnetic compounds into biomolecules without compromising its 3D-structure is a major challenge in organic and biochemistry. In principle there are different ways for post-synthetic functionalization of biomolecules. On the one hand, already available functional groups such as amino, carboxyl and hydroxyl can be addressed^8^. On the other hand, noncanonical amino acids^10^ or cysteines^6,33^ can be incorporated by genetic engineering during biosynthesis. Thereby, different binding sites are artificially created for selective reactions^7^. For example, thiol groups can be used to couple maleimide residues selectively under mild conditions^34^ equally as ethynyl^35^ and azido groups^36^. Artificial binding sites are outstanding specific targets for functionalization but are laborious, costly and do not guarantee the integrity of the molecule’s structural features. The better choice would be to use already existing functionalities with high specific targeting such as cysteine residues. However, it is exceedingly rare that proteins contain unpaired thiol groups^37^. In contrast, paired thiol groups, so-called disulfide bonds, are frequently present in therapeutically relevant proteins. Such disulfide bonds can easily be reduced to generate reactive thiol groups, which can be utilized as nucleophilic sites for a variety of modifications^38–41^.

The decisive advantage of addressing disulfide bridges in peptides or proteins is the possibility for site-selective labeling without compromising the conformational integrity of the substrate^42^. There are existing labels, such as bifunctional spin label, which fulfil this condition. Recently bis-MTSSL (trans-3,4-bis[[(methylsulfonyl)thio]methyl]-2,2,5,5-tetramethylpyrrolidin-1-yloxyl) was proposed as an effective and commercially available agent for a pH dependent reversible spin labeling by insertion into a disulfide bridge^43–46^. For those applications where a pH dependence reversibility of the spin-labeling is undesired or stability under reductive conditions is needed, it is necessary to employ a stronger binding scheme to attach the spin-label to a bioactive molecule. This necessitates a different insertion mode of the spin-label. The chemistry behind this approach relies on two sequential MICHAEL additions of the thiol groups to α,β-unsaturated carbonyl compounds with a leaving group in a suitable allylic position. The bis-alkylation conjugation reagents of Liberatore et al.^39^, relying on sulfinate leaving groups are especially successful as structure preserving intercalators. This has been shown initially for the site-selective introduction of radio labels^39^ and pegylations^38,47^. Later Weil et al.^40,48–51^ developed this chemistry further building an extensive library of intercalators as a versatile toolkit with different functionalities, which can be implemented into many biomolecules including antibodies^52^.

**Scheme 1:**
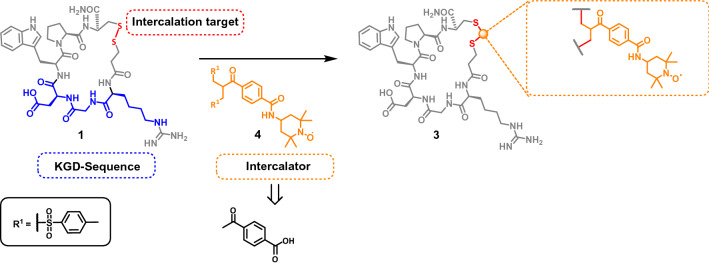
The cyclic structure and the "K"GD-amino acid sequence ("Lys"-Gly-Asp) of eptifibatide **1** with insertion of the synthesized bis-sulfone based spin label **4** into the reduced disulfide bond to obtain the spin labeled eptifibatide **3**. We followed the usual nomenclature of the binding motif although it should read homo-R-G-D instead of KGD^56,57^.

Herein we would like to describe the synthesis and incorporation of a bis-sulfone based spin label into the cyclic heptapeptide eptifibatide (Scheme 1). Eptifibatide (Integrilin) **1** occurs in the venom of certain rattlesnakes (s*istrurus miliarius barbourin*) and is used as an antiplatelet aggregation inhibitor in medicine. The high potential of this drug is based on its cyclic structure and the KGD amino acid sequence (Lys-Gly-Asp)^53–55^.

We will demonstrate by high performance liquid chromatography (HPLC) and electrospray ionization mass spectrometry (ESI–MS) that the selective incorporation of radicals into the disulfide bond is possible. Additionally, we will show by EPR that the radical activity is unaffected by the intercalation chemistry and by DNP experiments we demonstrate its potential application scope.

## Results and discussion

### Synthesis of the spin label and its intercalation in eptifibatide

**Scheme 2:**
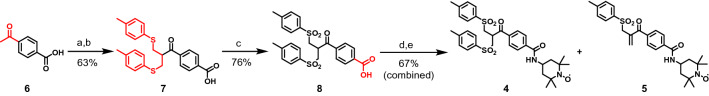
Synthesis of the intercalator mixture **4** and **5.** **(a)** Paraformaldehyde, dimethylamine hydrochloride, isopropanol, reflux, 24 h; **(b)** 4-methylthiophenol, isopropanol/H_2_O, RT, 24 h; **(c)** H_2_O_2_, AcOH, 30 °C, 24 h; **(d)** EDC·HCl, HOBt, CH_2_Cl_2_/*N*-methylmorpholine, 0 °C, 0.5 h; **(e)** 4-amino-TEMPO, CH_2_Cl_2_/*N*-methylmorpholine, 0 °C, 24 h.

With the exception of the last step the synthesis of the target compounds **4** and **5** was achieved according to the literature (Scheme 2)^58^.

After a double mannich type reaction the resulting intermediate was directly used in the subsequent substitution reaction with 4-methylthiophenol as a nucleophile yielding the bisthioether **7** in 63% combined yield after recrystallization. Oxidation of **7** by H_2_O_2_ in acetic acid delivered the bissulfonylated acid **8** which was coupled to 4-amino-TEMPO by 1-Ethyl-3-(3-dimethylaminopropyl)carbodiimid (EDC) mediated amide-coupling. After purification by column chromatography, the product was obtained as an inseparable mixture of bissulfone **4** and allylsulfone **5**. Considering the fact, that under the conditions of the intercalation chemistry **4** is converted to the active michael-acceptor **5** anyway, no attempt was made to separate both compounds.

**Scheme 3:**
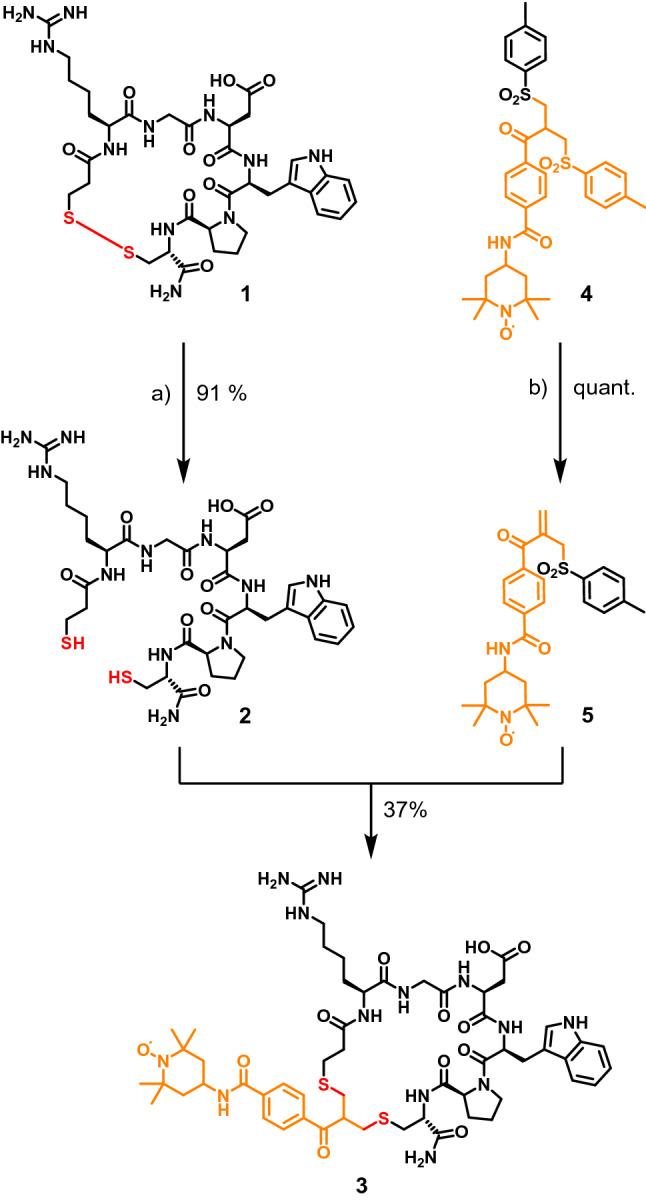
Intercalation of **5** into eptifibatide **1**. **(a)** Dithiothreitol, MeCN:H_2_O 1:1, RT, 20 h; **(b)** MeCN:H_2_O 1:1, pH 7.8 (NaH_2_PO_4_), RT, 24 h; **(c)** MeCN:H_2_O 1:1, pH 7.8 (NaH_2_PO_4_), RT, 24 h.

Having successfully prepared the target compound mixture **4/5**, we next introduced the spin-label into the disulfide bridge of eptifibatide. The two-step process required for this is illustrated in Scheme 3.

First eptifibatide **1** is reduced by dithiothreitol (DTT) to open the disulfide bond ^59^, which yields the bismercaptan **2**. After its purification by semi-preparative HPLC, it is used in the subsequent intercalation reaction. For that purpose, **2** is added to the allylsulfone **5,** which was prepared from its mixture with bissulfone **4** by mild base treatment. The intended double michael reaction occurs as expected delivering the final product **3** with 12% yield after semi-preparative HPLC.

### Proof of spin label insertion into eptifibatide

**Figure 1:**
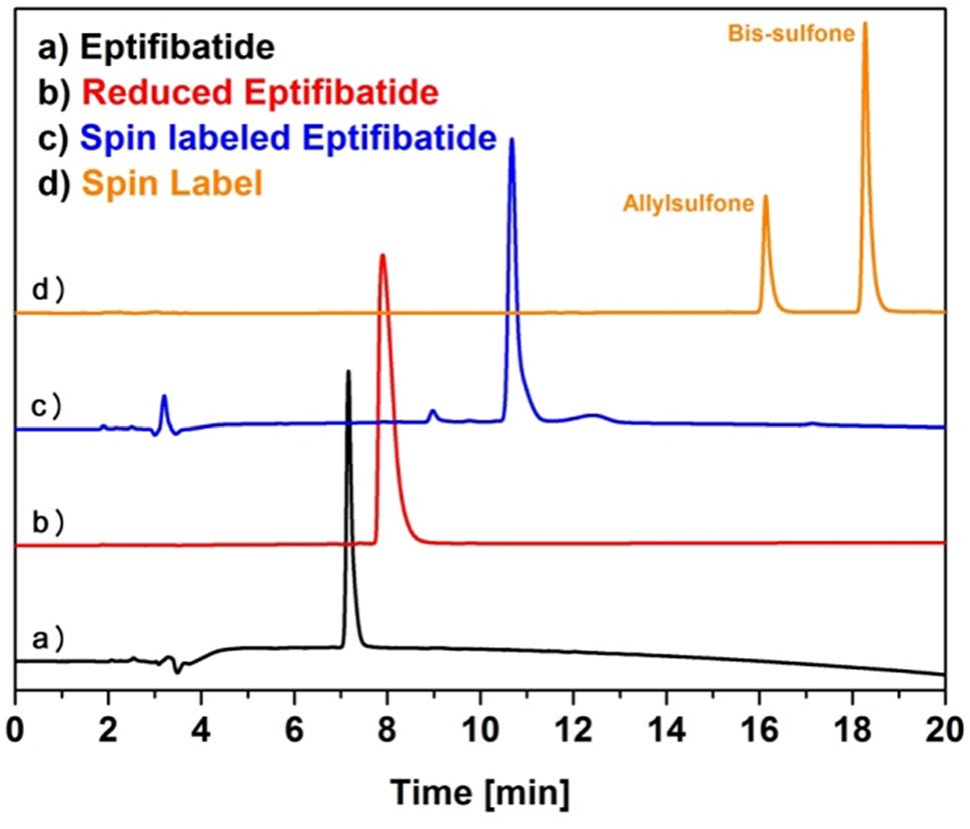
HPLC traces (214 nm, with gradient of acetonitril in water from 20 to 80% with 0.1% TFA for 20 min) illustrating the intercalation process. **(a)** eptifibatide **1**, **(b) **reduced eptifibatide **2**, **(c)** spin labeled eptifibatide **3,** **(d)** mixture of the spin labels **4** and **5**.

**Figure 2:**
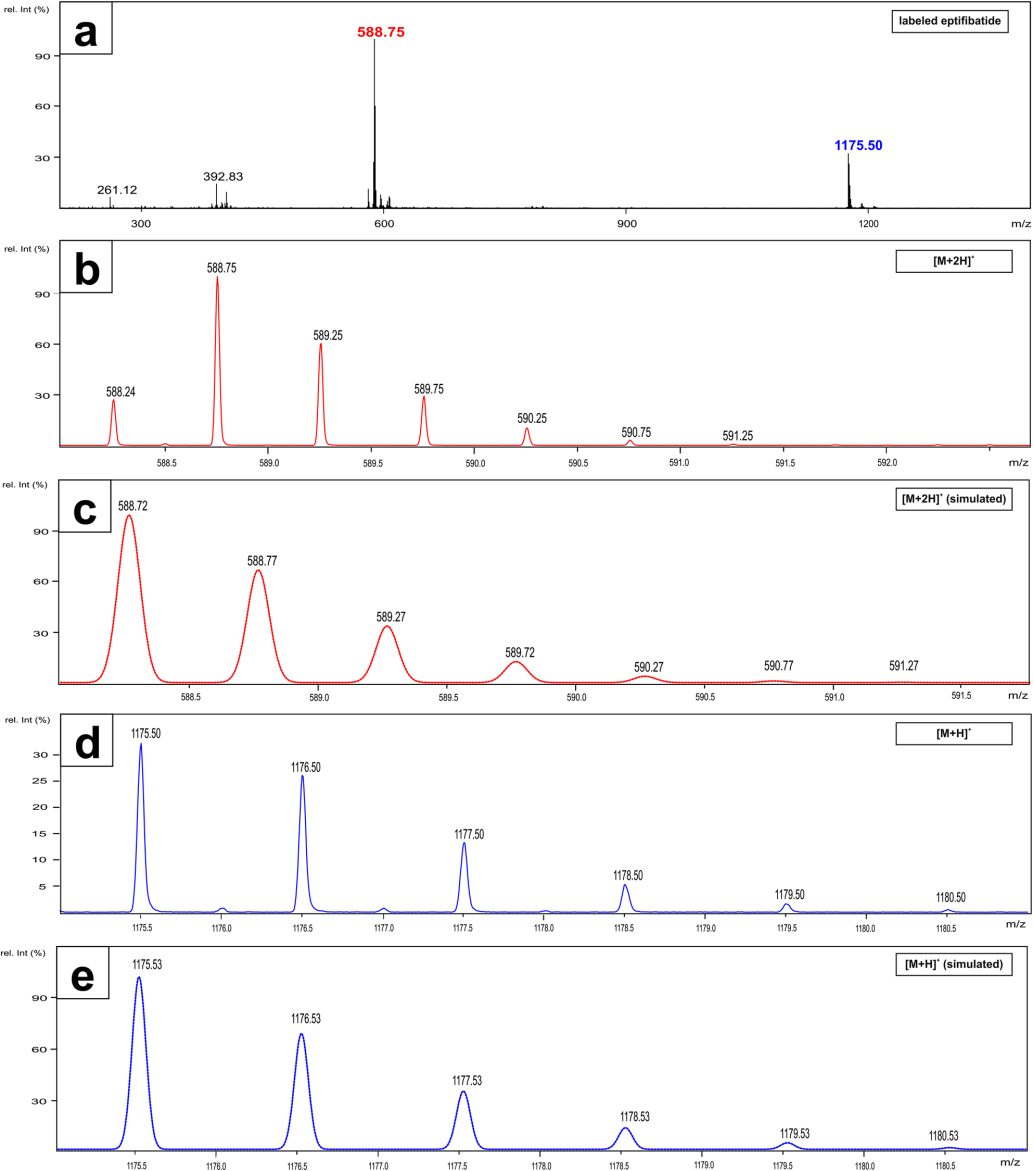
**(a)** ESI–MS of **3** displaying the [M + 2H]^2+^ and the [M + H]^+^ peaks. **(b)** Expansion of the [M + 2H]^2+^ peak (experimental). **(c)** Simulation of the [M + 2H]^2+^ peak. **(d)** Expanded experimental [M + H]^+^ peak. **(e)** Simulation of the [M + H]^+^ peak.

The covalent insertion of the spin label is confirmed by electrospray ionization mass spectrometry (ESI-MS) and HPLC. The insertion process can be followed by comparison of the HPLC traces for each step (Fig. 1) and is validated by the mass spectrum of the spin labeled compound **3** after product isolation (Fig. 2).

The HPLC trace of the spin label mixture displays the inseparable compounds of bissulfone **4** (t_R_ = 18.3 min) and allylsulfone **5** (t_R_ = 16.1 min) (Fig. 2d). This coexistence of the bis-sulfone based spin label and its activated species was validated by HPLC–MS (ESI Fig. 9). The intercalation process can be monitored unequivocally by comparison of the HPLC traces showing eptifibatide **1** with t_R_ = 7.2 min, reduced eptifibatide **2** with t_R_ = 7.9 min and labeled eptifibatide **3** with t_R_ = 10.7 min. (Fig. 1a–c).

In order to confirm the assignment of the signal at t_R_ = 10.7 min as labeled eptifibatide **3,** ESI–MS spectra were recorded and simulated (Fig. 2). As expected for **3,** the observed masses of 1175.5 m/z and 588.3 m/z clearly indicate the spin labeled eptifibatide molecular masses [M + 1H]^+^ and [M + 2H]^2+^.

**Figure 3:**
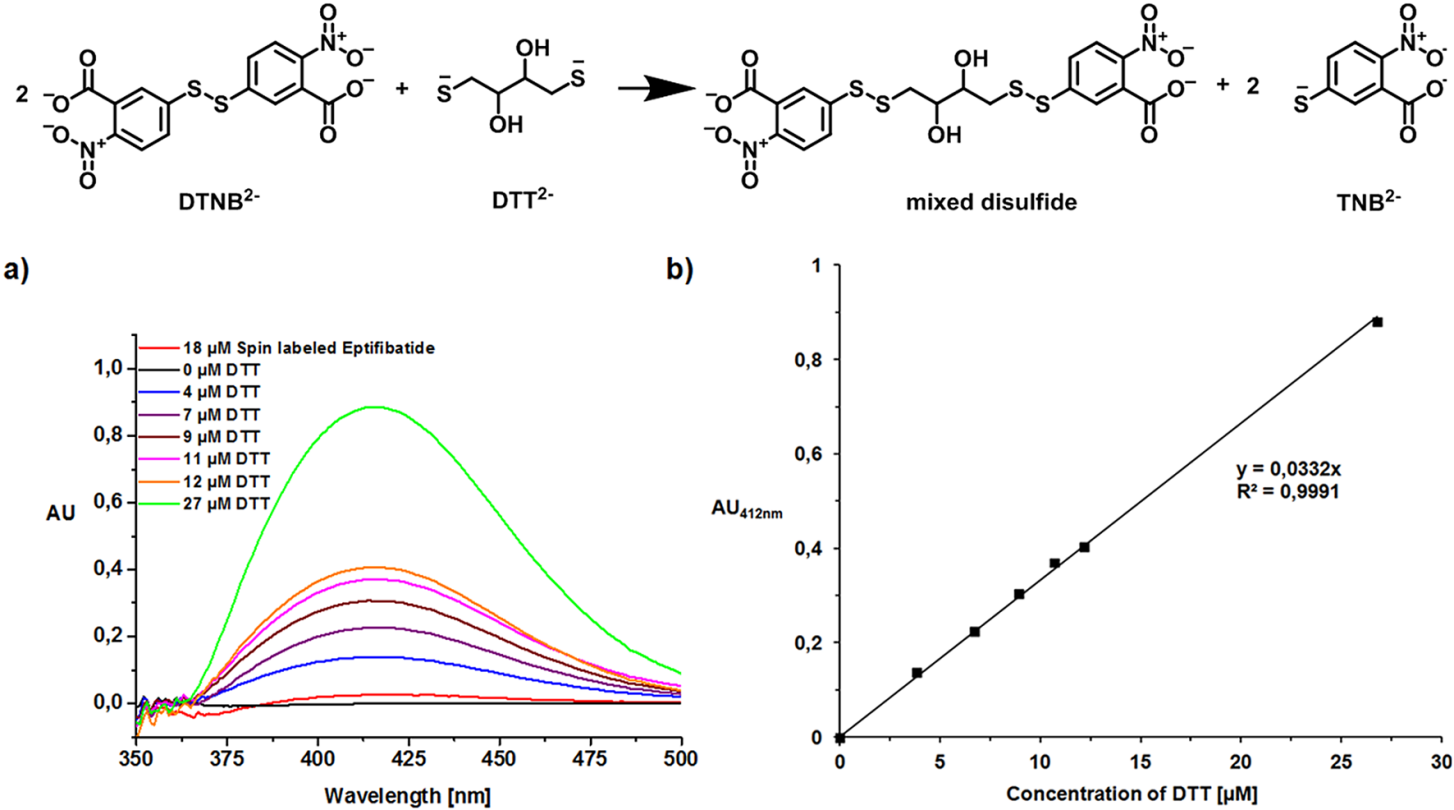
Reduction of Ellman’s reagent (DTNB) with dithiothreitol (DTT) (above). **(a)** Dithiothreitol (DTT) concentration dependent UV-spectra and the UV-spectrum of eptifibatide in the presence of Ellman’s reagent (DTNB). **(b)** Calibration curve of the absorbance UV light at 412 nm with known concentrations of dithiothreitol (DTT) after reduction reaction with Ellman’s reagent (DTNB).

As the singly and the doubly attached spin label have the same mass of 1175.5 *m/z*, the successful binding of the spin label to both thiol groups of eptifibatide cannot be determined by mass spectrometry. For this reason the amount of free thiol groups are determined by an Ellman’s test^60–62^, employing dithiothreitol (DTT) as standard and Ellman’s reagent (5,5'-Dithio-bis-(2-nitrobenzoic acid), DTNB). DTNB reacts with a free sulfhydryl group to yield a mixed disulfide and 2-nitro-5-thiobenzoic acid (TNB) (Fig. 3 above). The target of DTNB in this reaction is the conjugate base (R-S^−^) of a free sulfhydryl group. The molar extinction coefficient has a value of 14.150 M^−1^ cm^−1^ at 412 nm^60–62^. The extinction of TNB is not affected by changes in pH between 7.6 and 8. Sulfhydryl groups can be estimated in a sample by using known concentrations of thiol groups such as dithiothreitol (DTT), detecting their absorbance per UV–Vis (Fig. 3a) resulting in a calibration curve (Fig. 3b).

The Ellman’s test shows that at pH 8 the spin labeled eptifibatid consists of at least 90% complete inserted spin label by attaching to both thiol groups of eptifibatid. The resulting thiol-concentrations (see Table 1) are in very good agreement with the concentrations obtained employing previously published calibration curves of thiol-groups^60–62^.

**Table 1:** Thiol concentration of 18 µM spin labeled eptifibatid based on the calibration curve depicted in Fig. 3b or by exploiting the extinction coefficient of TNB (2-nitro-5-thiobenzoic acid) according to the literature^60–62^.

Method	Extinction coefficient of TNB at 412 nm (M^−1^∙cm^−1^)	Thiol concentration of 18 µM Eptifibatid (µM)
Own calibration curve	16.617	1.5 (8%)
Literature calibration curve	14.150	1.8 (10%)

In conclusion, we successfully synthesized a mixture of the bis-sulfone based spin label **4** together with its reactive elimination product **5** and we applied this mixture successfully to label the cyclic heptapeptide eptifibatide by site selective insertion of the label into its disulfide bridge.

### Proof of spin label activity after insertion

**Figure 4:**
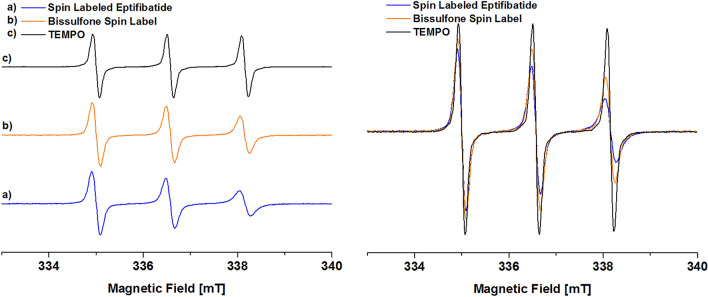
**(a)** EPR spectra of spin labeled eptifibatide **3** (blue), **(b)** bis-sulfone based spin label **4** (orange) and **(c)** TEMPO as reference (black). Note: Spectra are normalized by the maximum intensity (left side). Spectra are shown without normalization (right side). Experimental conditions were identical for all samples. Samples were measured at concentrations of 1 mM in DMSO at 293 K.

With the labeled eptifibatide **3** at hand, we next measured EPR spectra to verify its radical character as a precondition for the intended DNP measurements (Fig. 4).

**Figure 5:**
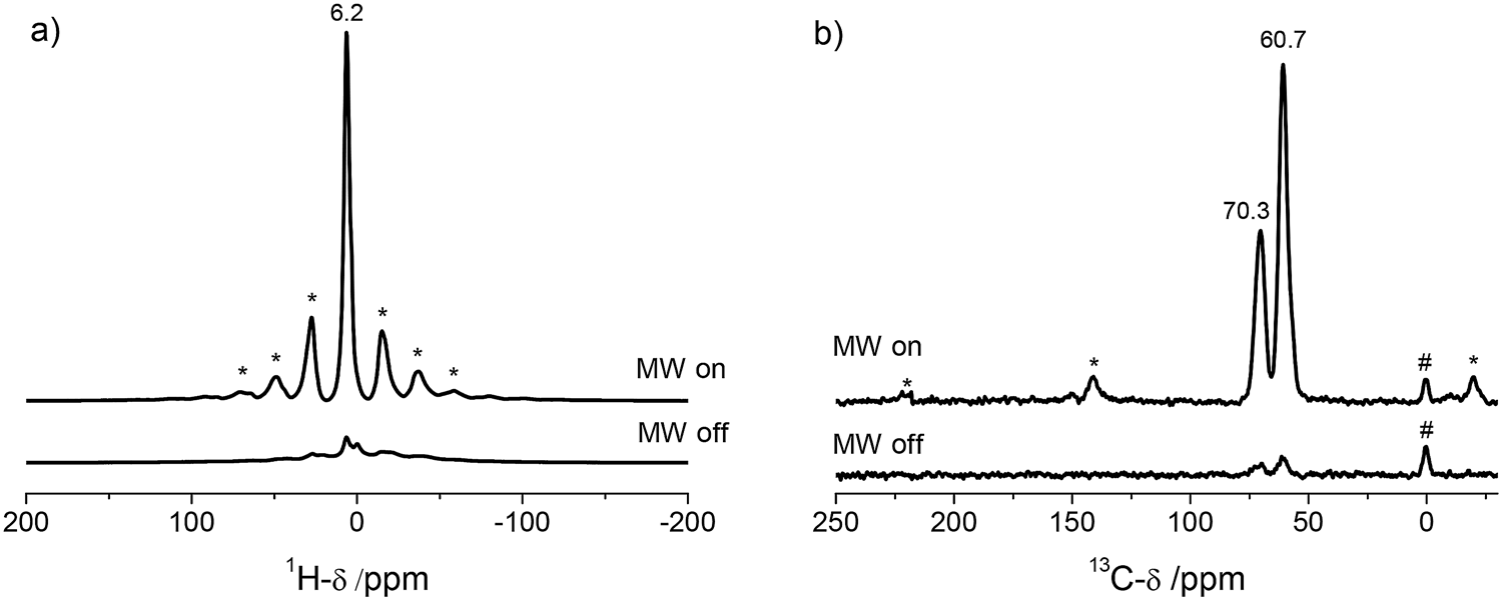
^1^H **(a)** and ^1^H^13^C CP MAS NMR **(b)** spectra of 15 mM spin labeled eptifibatide **3** in a glycerol-d_8_/D_2_O/H_2_O matrix with (MW on) and without (MW off) microwave irradiation measured at 9.4 Tesla at 8 kHz spinning rate. In the ^13^C spectra the two signals at 60.7 ppm and 70.5 ppm refer to the glycerol and the small signal at 0 ppm is attributed to the silicon plug marked with #, used to tightly close the liquid in the NMR rotor. The asterisks indicate the spinning side bands of the glycerol in the ^13^C spectra and the spinning sidebands of the water in the ^1^H spectra.

The EPR spectra of the labeled eptifibatide **3**, the spin label **4/5** and 4-amino-TEMPO reference show the expected triplet caused by hyperfine interaction between the unpaired electron spin and ^14^N nitrogen (S = 1) as it is typical for nitroxide radicals. Since an absolute quantification of the signals is not feasible using these experiments, we performed a semi quantitative analysis by double integration of the spectra. For this, we compared the absolute integrals of the EPR spectra lines of TEMPO, the bissulfone spin label and the spin labeled eptifibatide. With respect to TEMPO as a reference, this results in a relative activity of 90% for the bissulfone spin label and 79% for the spin labeled eptifibatide. Based on these results, we next investigated the DNP activity of the spin labeled eptifibatide **3** in a typical DNP solvent mixture glycerol-d_8_/D_2_O/H_2_O = 60/30/10 (v/v). To ensure, that the spin label is stable in this solvent mixture, HPLC analysis was performed. The comparison of the HPLC traces of the labeled eptifibatide in its neat form and in the above mentioned solvent mixture (see ESI Fig. 14) shows no significant differences, thus proving its stability under the measurement condition. To inspect the efficiency of the polarization transfer for DNP applications ^1^H MAS NMR (Fig. 5a) and ^1^H^13^C CP MAS NMR (Fig. 5b) spectra were recorded. By comparison of the ^1^H MAS spectra (Fig. 5a) with microwave irradiation (MW On) and without microwave irradiation (MW Off), it is observed that the signals under MW irradiation are enhanced by a factor of 14 as compared to the "MW Off" spectrum.

An even slightly higher signal enhancement is observed in the ^1^H^13^C CP MAS NMR spectrum of this sample for the signals of the carbons of glycerol at 60.7 and 70.5 ppm. Both signals are enhanced by a factor of 19 (Fig. 5b), which corresponds to a time saving factor of 361. This means that using this spin-label in solid-state DNP analytics will reduce the measurement time from one year to one day compared to standard solid-state NMR. The apparent discrepancy between the enhancement in ^1^H and ^1^H^13^C CP MAS NMR is most probably related to the inherent background signal from protons that are not polarized^63^.

Signals of eptifibatide are not detected in the spectrum, most probably due to its low concentration (ca. 15 mM), which is about 550 times smaller than the concentration of glycerol-d_8_ (8.2 M) in the DNP matrix. Furthermore, owing to the relatively small size of the eptifibatide, only the ^13^C nuclei of the glycerol may be visible in the spectrum due to signal bleaching. This phenomenon has been described in detail for other nitroxyl radical systems used in DNP NMR experiments ^64,65^ as well as for radical spin labels in protein systems^66^. To underline these hypotheses isotope labeling of the eptifibatide would be required, which is beyond the scope of this work.

## Conclusion

In this work, a new site selective bis-sulfone based spin label has been synthesized and its suitability for the introduction of a TEMPO-derived spin label by insertion into the disulfide bridge of a bioactive molecule has been demonstrated.

The new amino-TEMPO-modified intercalator was synthesized as a two-component mixture of the bis-sulfone **4** and its β-elimination product **5** in which both components can take part in the labeling reaction because the active michael acceptor **5** is produced by elimination under the reaction conditions. As an example for the feasibility of the intended intercalation process the spin label was inserted into the disulfide bridge of eptifibatide **3**, a synthetic cyclic heptapeptide derived from a disintegrin protein found in the venom of the rattlesnake s*istrurus miliarius barbourin*.

The preservation of the activity of the spin label after incorporation into eptifibatide’s disulfide bond was confirmed by EPR spectroscopy. Given the reductive conditions necessary for the insertion of the spin label into the disulfide bridge (DTT reduction) this is an important result^67^. Finally, solid-state DNP NMR measurements were carried out successfully, leading to DNP signal enhancements of 14 for the protons and 19 for the carbons of glycerol from the matrix, which corresponds to a time saving factor of up to 361.

While for the low-molecular weight eptifibatide **3** used here to demonstrate the principal feasibility of inserting spin-labels into S–S-bonds, only the hyperpolarized ^13^C-nuclei of the matrix were resolved in the ^13^C-NMR spectra, we assume that it is feasible to observe the nuclei of larger binding partners such as the activated platelet glycoprotein IIb/ IIIa receptor in binding studies^66,68^.

These encouraging results pave the way to the development and application of a powerful tool for the introduction of site selective radical spin labels into biomolecules and biosolids without compromising its conformational integrity for structural investigations employing solid-state DNP or advanced EPR techniques.

Furthermore, it should be possible to use this or similar compounds for HYPERSENSE dissolution DNP applications to investigate pharmacokinetics as a non-invasive method.

## Experimental section

### Synthesis

#### General

**Scheme 4:**
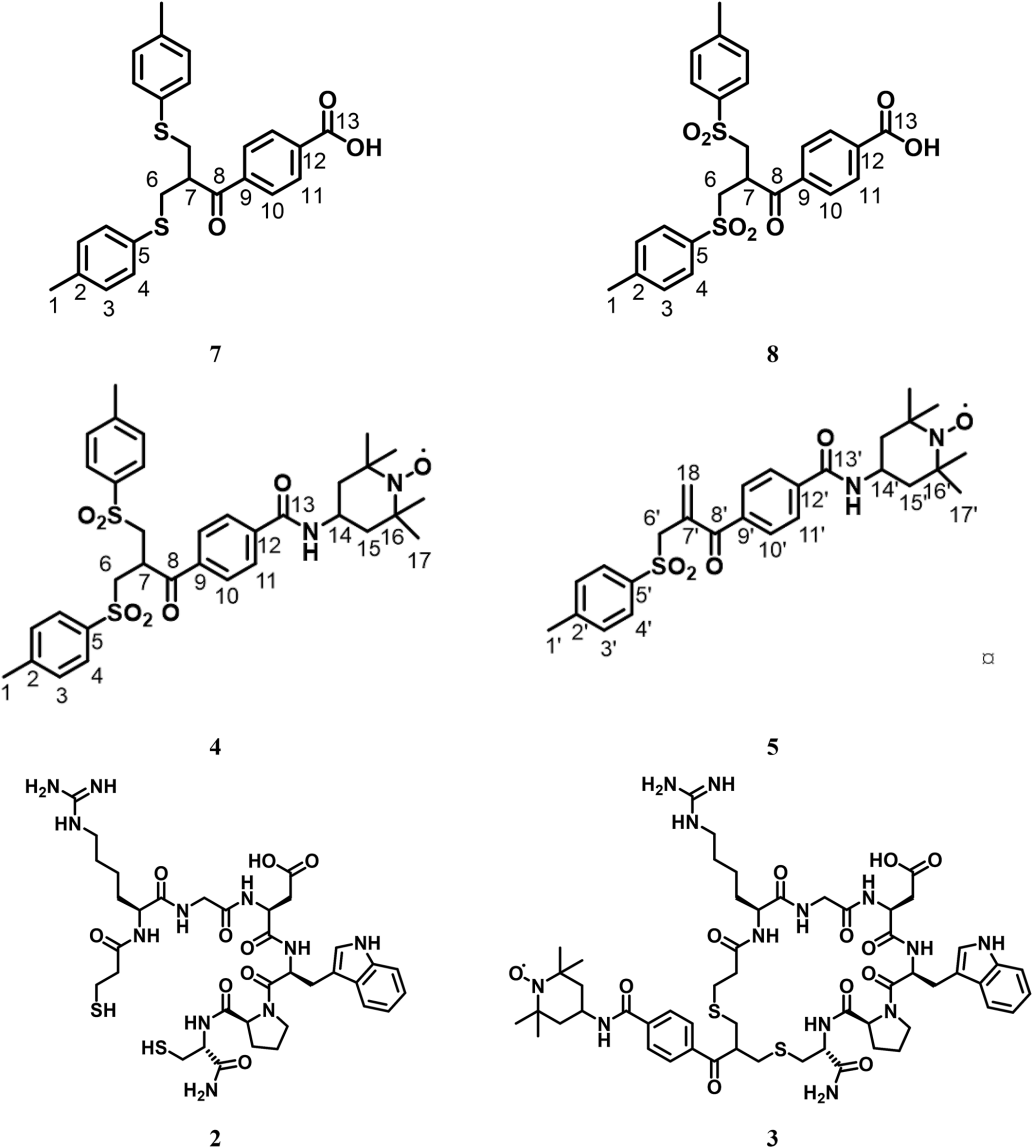
Chemical structures of compounds **2,3,4,5,7,8**.

All materials were purchased from commercial suppliers (Fisher Scientific, Acros Organics, Merck, ABCR, Alfa Aesar) and used without further purification unless otherwise described. Eptifibatide (CAS: 188627-80-7) was purchased from Ambeed, Inc. Solvents were purchased in HPLC grade and HOBt (1-Hydroxybenzotriazole) was dried by azeotropic codestillation with toluene. Deuterated solvents for NMR-spectroscopy were purchased from Sigma-Aldrich. All synthesized compounds are collected in Scheme 4.

### Thin layer chromatography

Thin layer chromatography was performed using silica gel (SilG/UV 252, plate thickness: 0.25 mm) by Macherey–Nagel GmbH & Co. KG. Visualization was achieved by UV fluorescence and quenching or oxidizing with KMnO_4_.

### Characterization

#### Liquid NMR

Solution-state NMR measurements have been conducted using a Bruker 300 MHz Avance III NMR spectrometer or 300 MHz Avance II NMR spectrometer (^1^H-NMR 300.16 MHz, ^13^C NMR 125.78 MHz) at the service department of the TU Darmstadt. The chemical shifts are given in ppm. As internal standard the chemical shift of the solvent peak was used.

### Reverse phase high-performance liquid chromatography (RP-HPLC)

Reversed phase high-performance liquid chromatography (RP-HPLC) for analytical purposes was conducted using a Waters HPLC setup consisting of a Waters Alliance e2695 equipped with a Waters 2998 PDA detector. The detection wavelength was chosen depending on the analyte between 214, 254, 280 and 301 nm. The eluent system for the HPLC system comprised eluent A (0.1% aq. TFA) and eluent B (99.9% acetonitrile containing 0.1% TFA). Unless otherwise specified analytical HPLC runs were conducted at a flow rate of 2 ml/min with an eluent gradient from 20 to 80% of eluent B in eluent A over 20 min. For analysis a Nucleosil 100–5 C18 column from Macherey–Nagel (5 µm, 100 Å) was used. Preparative purification of the peptide was performed on a Knauer Multokrom RP18 column 20 × 250 mm (5 μm, 100 Å) employing a flow rate of 9 mL/min and an acetonitril gradient from 0 to 50% without TFA in water over the course of 60 min.

### Ellman’s test

Ellman’s test quantifies free thiol groups according to the literature^60–62^. DTNB (5,5'-Dithio-bis-(2-nitrobenzoic acid, Ellman’s reagent) reacts with free sulfhydryl groups to yield a mixed disulfide and 2-nitro-5-thiobenzoic acid (TNB). Thus the absorbance of TNB can be detected at 412 nm. In this connection dithiothreitol (DTT) is used as a standard for calibration.

A 20 mM DTNB stock solution was prepared by dissolving 8 mg DTNB in 1 ml DMSO. Dilution of the stock solution 100 fold with 0.1 M disodium hydrogen phosphate dihydrate (pH 8.0) results in 0.2 mM DTNB working solution. The reduction reaction of DTNB was achieved by adding 510 μl of 0.2 mM DTNB work solution to each 1 ml centrifuge tube, adding 50 μl of samples with known concentrations in DMSO and mixing by vortexing. The blank was set by adding 50 μl DMSO to 510 μl of 0.1 mM DTNB work solution. The incubation time was about 10 min at room temperature. The absorbance was measured with a J & M Analytik AG TIDAS S spectrometer against blank at 412 nm.

### Electrospray mass spectrometry (ESI–MS)

ESI (electron spray ionization) mass spectra were recorded with a Bruker Esquire-LC mass spectrometer at the service department of the TU Darmstadt.

### Electron paramagnetic resonance (EPR)

The EPR spectra for all samples were measured on an EPR Miniscope MS-400 (Magnettech, Germany) equipped with an A TC H03 temperature controller and a rectangular TE102 resonator operating at 9.43 GHz. All EPR spectra were recorded with modulation amplitude of 0.1 mT and a modulation frequency of 100 kHz. Spectra were taken under 24 dB of microwave attenuation with a mantissa gain of 1 and an exponential gain of 1. Measurements were performed at 298 K. A magnetic field range of 14 mT was swept with a center B_0_ field of 337 mT with a sweeping time of 60 s to acquire 2048 points.

### Dynamic nuclear polarization (DNP) NMR experiments

All measurements were performed on a Bruker Avance NEO 400 MHz DNP spectrometer equipped with a 4.8 T Bruker gyrotron system operating at second harmonics generating microwaves (MW) at a frequency of 263 GHz. A 3.2 mm low temperature triple resonance probe was utilized in ^1^H/^13^C/^15^ N configuration leading to a center frequency of 400.2 MHz for ^1^H and 100.6 MHz for ^13^C. Sample temperatures of 97 K or 107 K for measurements without or with MW irradiation, respectively, were nominally noted. The difference in the temperature is caused by the MW irradiation at a fixed maximum cooling power. A MAS spinning frequency of 8 kHz was applied for all ^1^H and ^1^H → ^13^C CP MAS.

^1^H MAS measurements were performed with a π/2 excitation pulse of 2.3 µs and a recycle delay of 5 s. 4 scans were applied. The spectra were referenced to TMS by setting the signal of the silicon plug to 0 ppm.

^1^H → ^13^C CP MAS experiments were recorded with a contact time of 2 ms. The recycle delay was set 18.2 s and 8 scans were applied. The recycle delay was set to 1.3 times T_1_ of the protons, which was measured with a T_1_ built-up experiment using a saturation recovery pulse sequence. The ^13^C spectra were referenced relative to the signal of the silicon plug (–Si–CH_3_ 0 ppm). For decoupling of protons the spinal-64 pulse sequence was used^69^.

### Synthesis of the bis-sulfone based spin label

#### Synthesis of bisthioether 7

According to literature ^48^ 5.66 g (34.48 mmol, 1.00 eq.) 4-Acetylbenzoicacid, 5.01 g (167.0 mmol, 4.84 eq.) paraformaldehyde and 13.62 g (167.0 mmol, 4.84 eq.) dimethylamine hydrochloride were diluted in iso-propanol and heated to reflux for 24 h. Upon cooling a precipitate appears, which was resolved by adding H_2_O. After adding 8.56 g (68.95 mmol, 2.00 eq.) 4-Methylbenzenethiol an Ar-atmosphere was applied. The reaction was heated to reflux again for 24 h and the precipitated solid was filtered after cooling to room temperature. The solid was rinsed with water, dried and recrystallized in methanol to obtain the product as a colorless solid.

#### Yield

9.47 g (63%); ^**1**^**H-NMR** (300 MHz, CDCl_3_, 301.2 K): δ (ppm) = 11.11 (bs, 1-COOH), 8.06 (d, 3 J = 8.3 Hz, 2-H10), 7.63 (d, 3 J = 8.3 Hz, 2-H11), 7.15 (d, 3 J = 7.98 Hz, 4-H4), 7.06 (d, 7.98 Hz, 4-H3), 3.83 (p, 1-H7), 3.22 (m, 4-H6), 2.36 (s, 6-H1); ^**13**^**C-NMR** (75 MHz, CDCl_3_, 301.2 K): δ (ppm) = 200.0 (8-C), 170.9 (13-C), 140.5 (2-C), 137.2(9-C), 132.8 (12-C), 131.5 (3-C), 131.0 (5-C), 130.2 (11-C), 129.8 (4- C), 128.3 (10-C), 45.9 (7-C), 36.3 (6-C), 21.1 (1-C); **EA** (C_25_H_24_O_3_S_2_): calculated: C: 68.78 H: 5.54 N: 0.00, measured: C: 68.95 H: 5.47 N: 0.00; **M**_**p**_**.**: 137.5–140.0 °C (Scheme 4).

#### Oxidation of bisthioether 7 to bissulfone 8

3.00 g (6.87 mmol, 1.00 eq.) of the bisthioether **7** were dissolved in 100 mL acetic acid and heated to 30 °C. ^58^ After adding 4.56 mL (44.67 mmol, 6.50 eq.) H_2_O_2_ (30–35%) the resulting mixture was stirred overnight at that temperature. Diethylether (50 mL) and water (100 mL) were added and the phases were separated. The aqueous phase was extracted additional two times and the combined organic phases were washed with a saturated solution of NaHSO_3_ and brine. The organic phase was dried over Na_2_SO_4_, filtered and the solvent removed under reduced pressure. The crude product was recrystallized in water. A colorless solid was obtained.

#### Yield

2.60 g (76%), ^**1**^**H-NMR** (300 MHz, DMSO-d_6_, 303 K): δ (ppm) l = 13.35 (bs, 1-COOH), 7.95 (d, 2-H10, 3 J = 8.63 Hz), 7.60–7.50(m, 6-H4, 11), 7.43(d, 2-H3, 3 J = 8.39 Hz), 3.99 (p, 1-H7), 3.89–3.65 (m, 4-H6), 2.45 (s, 6-H1); ^**13**^**C-NMR** (75 MHz, CDCl3, 301.2 K): δ (ppm) 195.5 (8-C), 166.3 (13-C), 145.1 (2-C), 137.8 (9-C), 135.9 (5-C), 130.1 (3-C), 129.4 (10-C), 128.2 (11-C), 127.8 (4-C), 54.9 (6-C), 35.2 (7-C), 20.9 (1-C); EI-MS: 344 (5, [M-C_7_H_7_O_2_S] +), 149 (100, [M-C_17_H_19_O_4_S_2_] +), 91 (95, [M-C_18_H_17_O_7_S_2_] +); **EA** (C_25_H_24_O_3_S_2_): calculated: C: 59.98 H: 4.83 N: 0.00, measured: C: 59.96H: 4.89 N: 0.00; **M**_**p**_**.**: 169.0–172.5 °C.

### Synthesis of bis-sulfone based spin labels 4 and 5

In a 100 mL round bottom flask 500 mg (0.99 mmol, 1.00 eq.) of bis-sulfone **8** and 0.22 mL (1.99 mmol, 2.00 eq.) *N*-methylmorpholine were dissolved in 25 mL methylene chloride and cooled to 0 °C. After adding 148.5 mg (1.099 mmol, 1.10 eq.) HOBt and 229.8 mg (1.20 mmol, 1.20 eq.) EDC•HCl the reaction was stirred at 0 °C until TLC shows complete conversion. 171 mg (0.99 mmol, 1.00 eq.) 4-Amino-TEMPO were added and stirred overnight, while warming up to room temperature. To isolate the product, water (50 mL) was added and the phases were separated. The aqueous phase was extracted additional two times with methylene chloride and the combined organic phases were washed with water and brine. Na_2_SO_4_ was added to the organic phase, filtered and solvent removed under reduced pressure. The crude product was purified by flash chromatography on silica (EA:PE/2:1). An orange foam was obtained, which was a mixture of bis-sulfone **4** and allylsulfone **5**.

#### Yield

437 mg (67%); **R**_**f**_ (EA:PE/2:1): 0.36 (allylsulfone), 0.40 (bis-sulfone); ^**1**^**H-NMR** (300 MHz, MeOH-d_4_, 303 K, equimolar amounts of ascorbic acid added): δ (ppm) = 7.92 (d, 2-H10’,3* J* = 8.43 Hz), 7.82 (d, 2-H10, 3* J* = 8.59 Hz ), 7.75 (d, 2-H11’,3* J* = 8.43 Hz) 7.65 (d, 4-H4, 3* J* = 8.31 Hz), 7.59 (d, 2-H11, 3* J* = 8.59 Hz, 7.45 (d, 4-H3, 3* J* = 8.31 Hz), 6.19 (s, 1-H18), 5.99 (s, 1-H18), 4.50 (d, 1-NH), 4.48 (m [overlapping], 1-H14), 4.31–4.23 (m, 1-H7), 3.83–3.76 (m, 4-H6, 6’), 3.69–3.63 (m, 4-H6, 6’), 2.52 (s, 6-H1, 1’), 2.01–1.95 (m, 3-H15, 15’), 1.76–1.66 (m, 3-H15, 15’), 1.32 (d, 18-H17, 17’); ^**13**^**C-NMR** (75 MHz, MeOH-d_4_, 303 K, equimolar amounts of ascorbic acid added): δ (ppm) = 196.7 (8-C), 173.5 (13-C), 147.1 (5-C), 146.5 (12-C), 138.2 (9-C), 136.4 (2-C), 130.1 (3-C), 128.7 (11-C), 128.1 (4-C), 127.8 (10-C), 61.5 (16-C), 56.4 (6-C), 44.5 (15-C), 42.4 (14-C), 37.9 (7-C), 30.9 (17-C), 20.4 (1-C); **HPLC: 4** t_R_ = 16.1 min and **5** with t_R_ = 18.3 min**; EI-MS**: 246 (70, [M-C_24_H_31_O_4_]^+^), 172 (35, [M-C_25_H_24_O_6_S_2_]^+^), 123 (65, [M-C_18_H_17_O_7_S_2_]^+^), 91 (100, [M-C_27_H_34_N_2_O_7_S_2_]^+^); **EI-HRMS** (C_28_H_27_NO_6_S_2_): measured: 653.2357 [M + H]^+^ (bis-sulfone), calculated: 653.2349 [M + H]^+^ (allylsulfone), measured: 497.2111 [M + H]^+^, calculated: 497.2105 [M + H]^+^ (allylsulfone); **IR** : ṽ = 1654, 1534, 1317, 1142 569 cm^-1^.

### Introducing spin label into the disulfid bond of eptifibatide

#### Reduction of eptifibatide 1 to dithiol 2

28 mg (33 µmol, 1.00 eq.) of eptifibatide **1** was dissolved in 5.0 mL acetonitrile and 5 mL anal. pur. water. After adding 20.6 mg (132 µmol, 4.00 eq.) of Dithiothreitol the mixture was stirred for 21 h at room temperature. Additional 10.2 mg (66 µmol, 2.00 eq.) Dithiothreitol were added before stirring for 2 more hours at room temperature. The solvent was removed under reduced pressure and the crude product was purified by HPLC.

#### Yield

25.6 mg; **HPLC:** t_R_ = 7.9 min; **EI-MS**: [M]^+^  = 834.34 m/z (simulated 834.34 m/z).

### Reaction of dithiol 2 to labeled eptifibatide 3

According to literature ^48^14.4 mg (22 µmol, 2.00 eq.) of the mixture label **4** and **5** was dissolved in 3.9 mL acetonitrile and 6 mL phosphate buffer (pH = 7.8, c = 50 mM) for 24 h at room temperature. After adding 9.00 mg (11 µmol, 1.00 eq.) reduced eptifibatide **2** in 1.8 mL MeCN and 2.7 mL phosphate buffer the mixture was stirred for additional 24 h. The solvent was removed under reduced pressure and the crude product purified by HPLC.

#### Yield

4.8 mg (37%); **HPLC:** t_R_ = 10.7 min; **EI-MS**: [M]^+^  = 1175.5 m/z (simulated 1175.5 *m/z*).

## Supplementary Information

Supplementary Information.
