# Supplementary material for: A novel strategy for site selective spin-labeling to investigate bioactive entities by DNP and EPR spectroscopy
Source: Sci Rep. 2021 Jul 1;11:13714. doi: 10.1038/s41598-021-92975-6 (PMC8249612; doi:10.1038/s41598-021-92975-6)
Supplement: Supplementary file 1 — Supplementary Information. [file 41598_2021_92975_MOESM1_ESM.pdf]

Electronic Supplementary Information

A Novel Strategy for Site Selective Spin-Labeling to Investigate Bioactive Entities by DNP and EPR Spectroscopy

Kevin Herr[a+], Max Fleckenstein[b+], Martin Brodrecht[a], Mark V. Höfler[a], Henrike Heise[c,d], Fabien Aussenac[e], Torsten Gutmann[a], Michael Reggelin[b*] and Gerd Buntkowsky[a*]

[a] Institute of Physical Chemistry, Technical University Darmstadt, Alarich-Weiss-Straße 8, D-64287 Darmstadt, Germany

[b] Institute of Organic Chemistry, Technical University Darmstadt, Alarich-Weiss-Straße 4, D-64287 Darmstadt, Germany

[c] Institute of Complex Systems, Structural Biochemistry (ICS-6), Forschungszentrum Jülich, D-52425 Jülich, Germany.

[d] Institut für Physikalische
Biologie, Heinrich-Heine-Universität Düsseldorf, D-40225 Düsseldorf, Germany

[e] Bruker France SAS, 34 rue de l'industrie , F-67160 Wissembourg, France

[+] These authors contributed equally to this work.

* eMail: gerd.buntkowsky@chemie.tu-darmstadt.de
re@chemie.tu-darmstadt.de

- confidential -

Contents

^{1}H liquid NMR spectra

^{13}C liquid NMR spectra

HPLC chromatograms

MS spectra

EPR spectra

1. Synthesis of bis-sulfone based spin label
   - 1.1 Bisthioether **7**
   - 1.2 Bis-sulfone **8**
   - 1.3 Bis-sulfone based spin label **4** and **5**

2. Synthesis of spin labeled eptifibatide
   - 2.1 Reduced eptifibatide **2**
   - 2.2 Spin labeled eptifibatide **3**

- confidential -

1 Synthesis of the bis-sulfone based spin label

1.1 Bisthioether 7

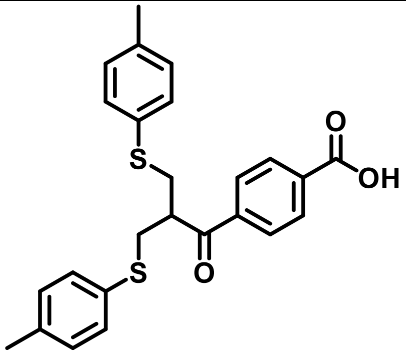

7

1.1.1 ^{1}H liquid NMR spectrum

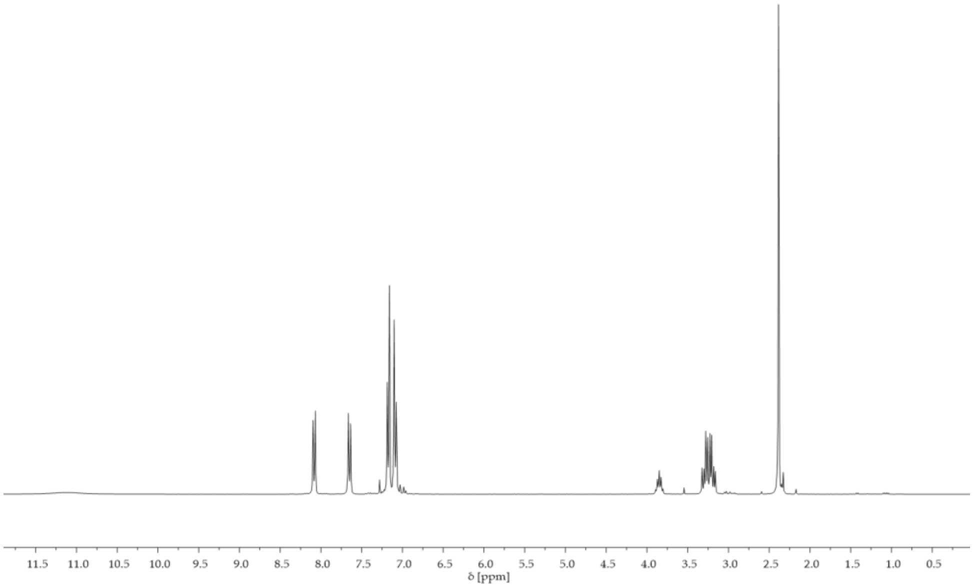

Figure 1: ^{1}H-NMR spectrum of bisthioether **7** in $CDCl_3$ at 301.2 K and 300 MHz.

1.1.2 ^{13}C liquid NMR spectrum

- confidential -

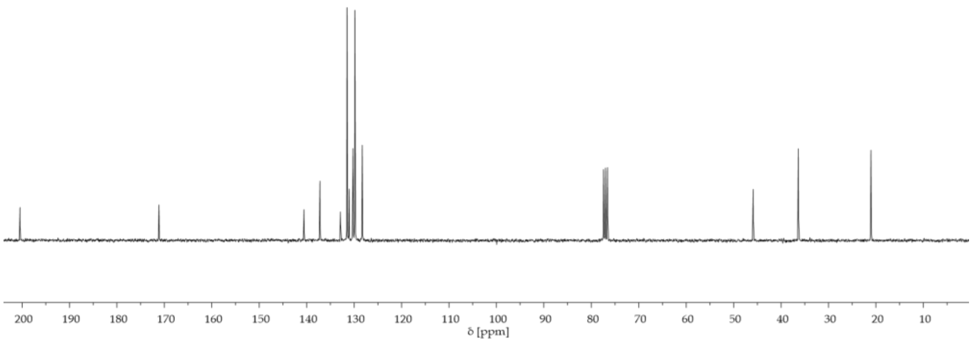

Figure 2: ^{13}C-NMR spectrum of Bisthioether **7** in $CDCl_3$ at 301.2 K and 75 MHz.

1.2 Bis-sulfone 8

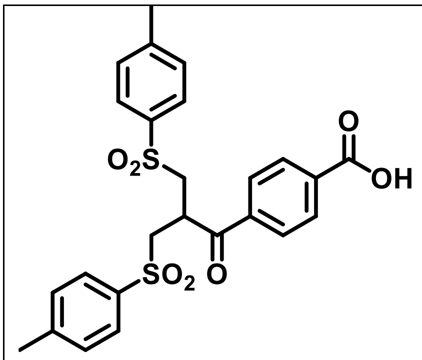

8

1.2.1 ^{1}H liquid NMR spectrum

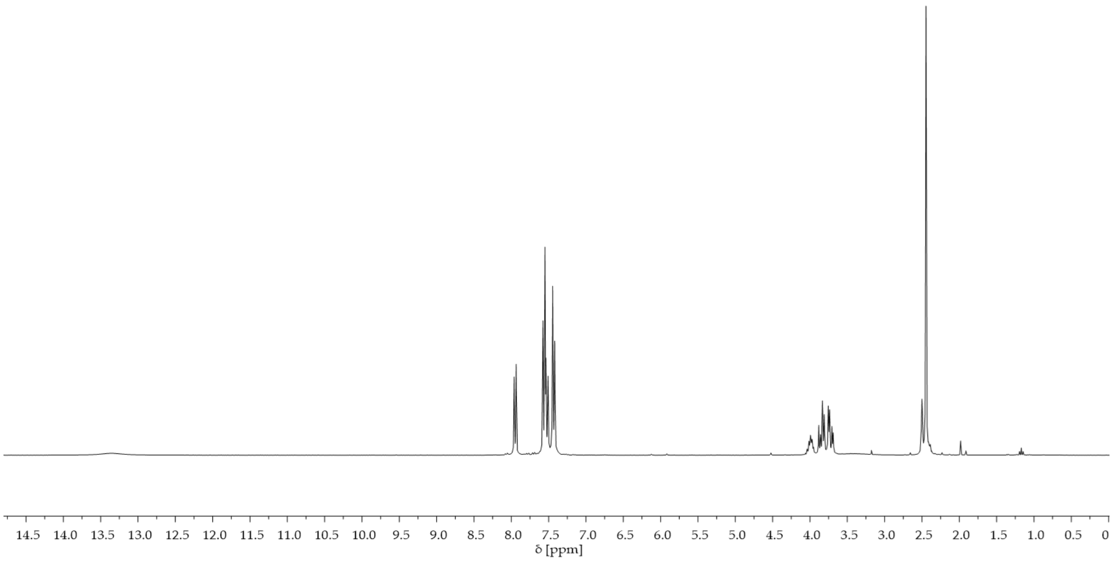

Figure 3: ^{1}H-NMR spectrum of bis-sulfone **8** in DMSO-d_6 at 303 K and 300 MHz.

- confidential -

1.2.2 ^{13}C liquid NMR spectrum

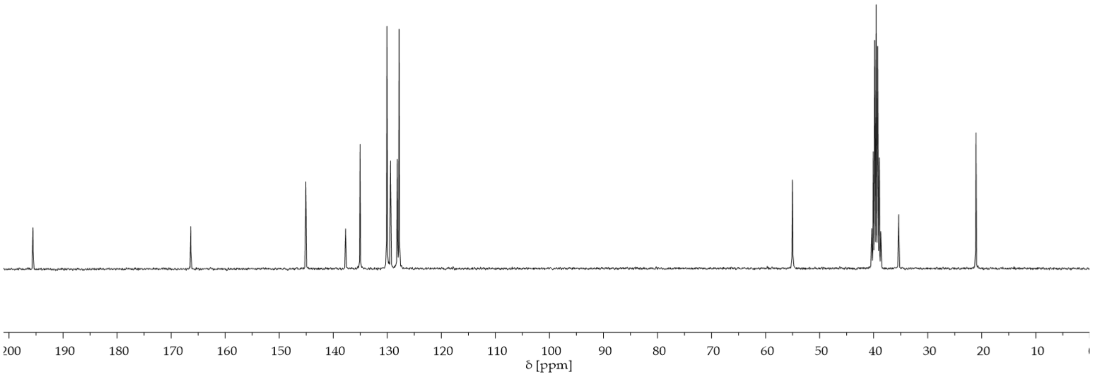

Figure 4: ^{13}C-NMR spectrum of bis-sulfone **8** in DMSO-d_6 at 303 K and 75 MHz.

1.3 Bis-sulfone based spin label 4 and 5

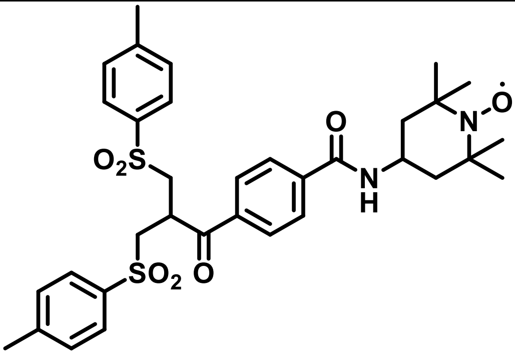	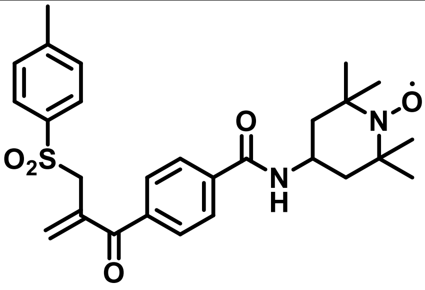
**4**	**5**

1.3.1 ^{1}H liquid NMR spectrum

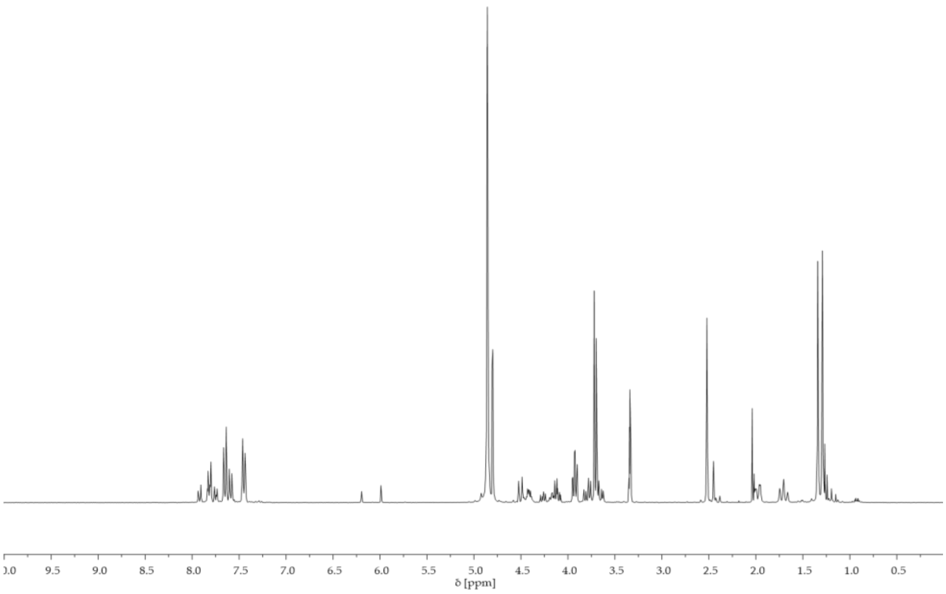

Figure 5: ^{1}H-NMR spectrum of the mixture of bis-sulfone based spin labels **4** and **5** (after addition of ascorbic acid) in MeOH-d_4 at 303 K and 300 MHz.

1.3.2 ^{13}C liquid NMR spectrum

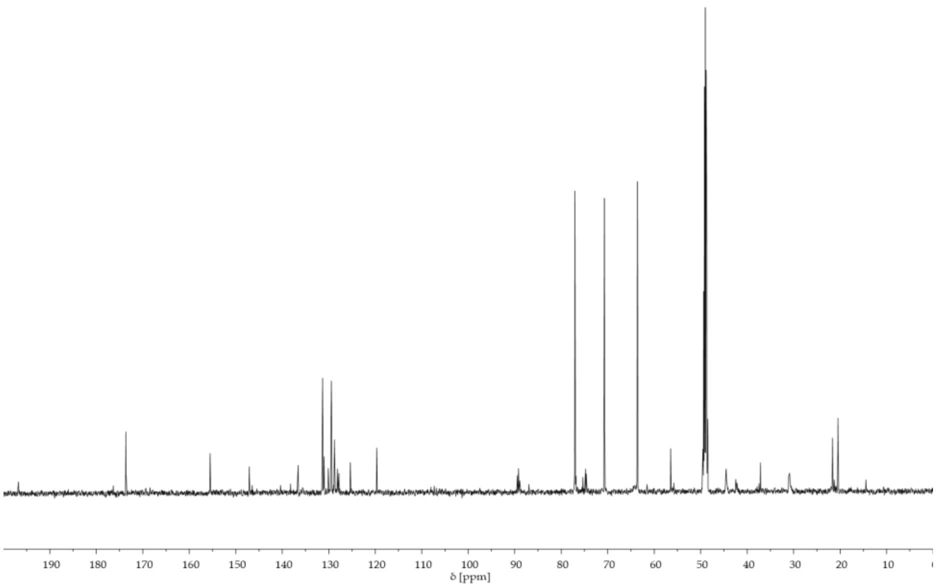

Figure 6: ^{13}C-NMR spectrum of the mixture of bis-sulfone based spin labels **4 and 5** (after addition of ascorbic acid) in MeOH-d_4 at 303 K and 75 MHz.

1.3.3 HPLC chromatogram

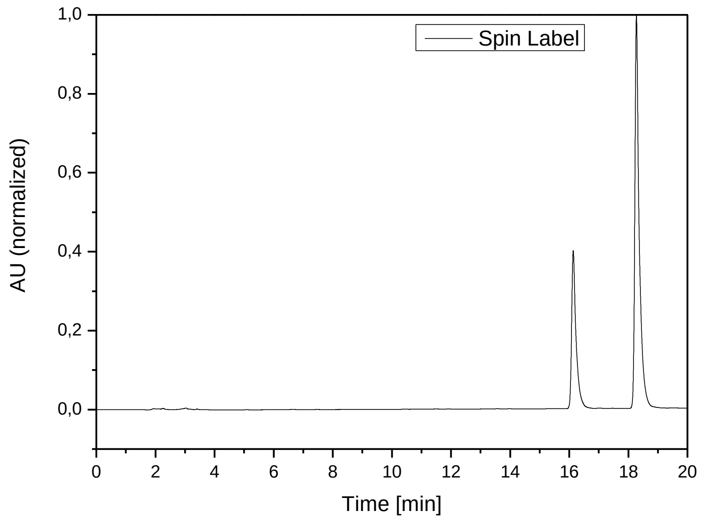

Figure 7: HPLC chromatogram of bis-sulfone based spin labels **4 (**t_R = 18.3 min.) and **5** (t_R = 16.1 min.) at 214 nm, with gradient of acetonitril in water from 20% to 80% with 0.1% TFA for 20 minutes.

1.3.4 EPR spectrum

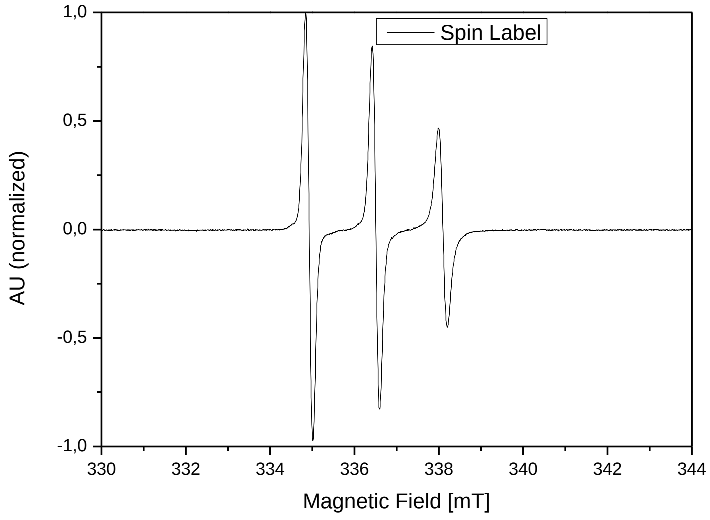

Figure 8: ESR spectrum of ca. 15 mM bis-sulfone based spin label **4** and **5** at 20 °C.

1.3.3 HPLC-MS chromatogram

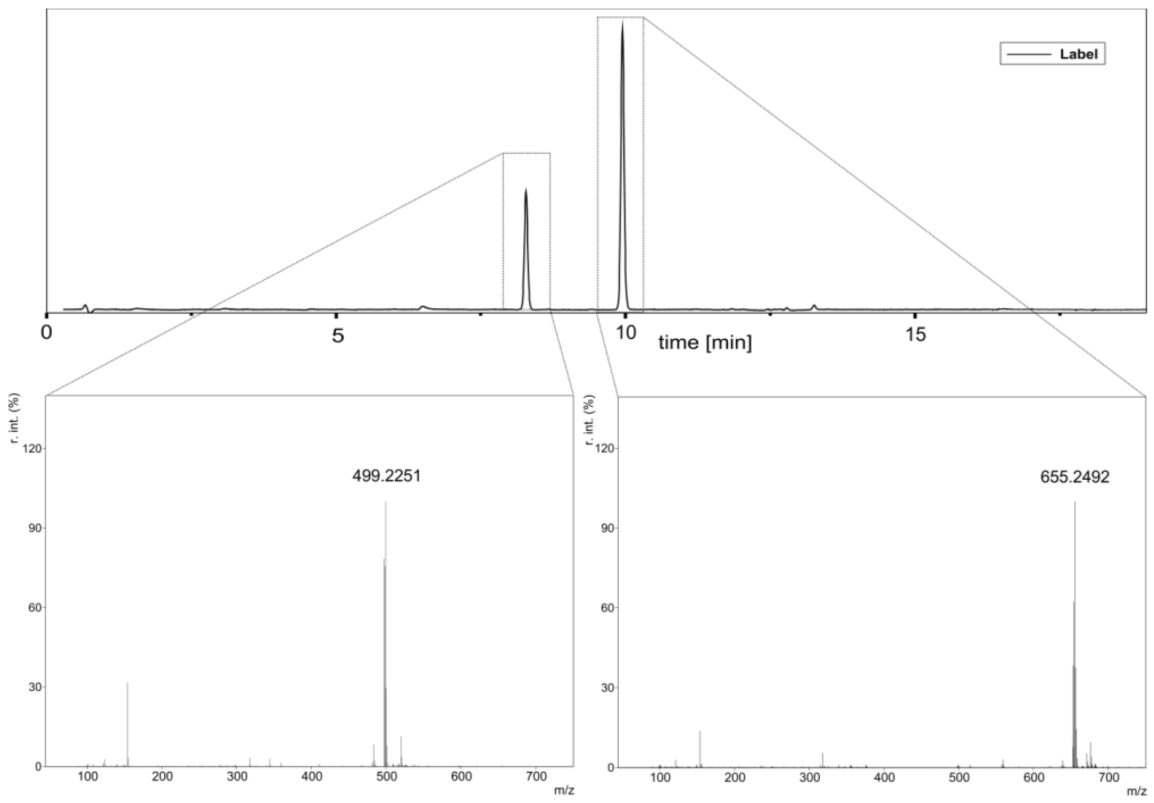

Figure 9: HPLC-MS chromatogram of bis-sulfone based spin label **4** and **5** at 180-400 nm, with gradient of acetonitril in water from 20% to 90% with 0.1% formic acid for 19 minutes.

2. Synthesis of spin labeled eptifibatide

2.1 Reduced eptifibatide 2

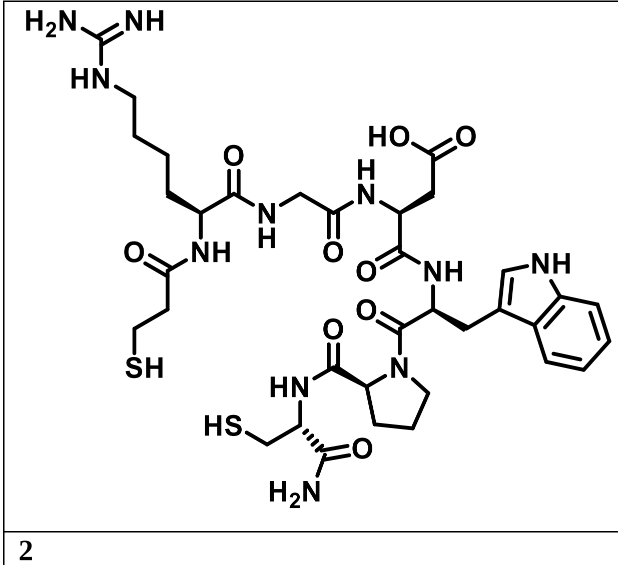

2

- confidential -

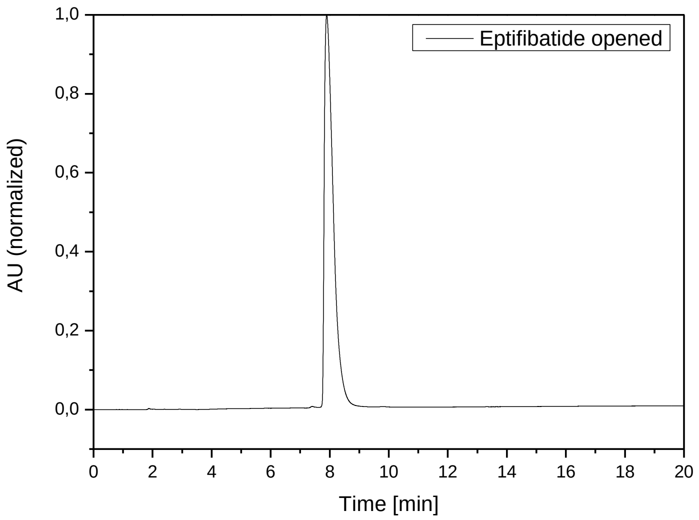

Figure 10: HPLC chromatogram of reduced eptifibatide **2** detected at 214 nm, with gradient of acetonitril in water from 20% to 80% with 0.1% TFA for 20 minutes.

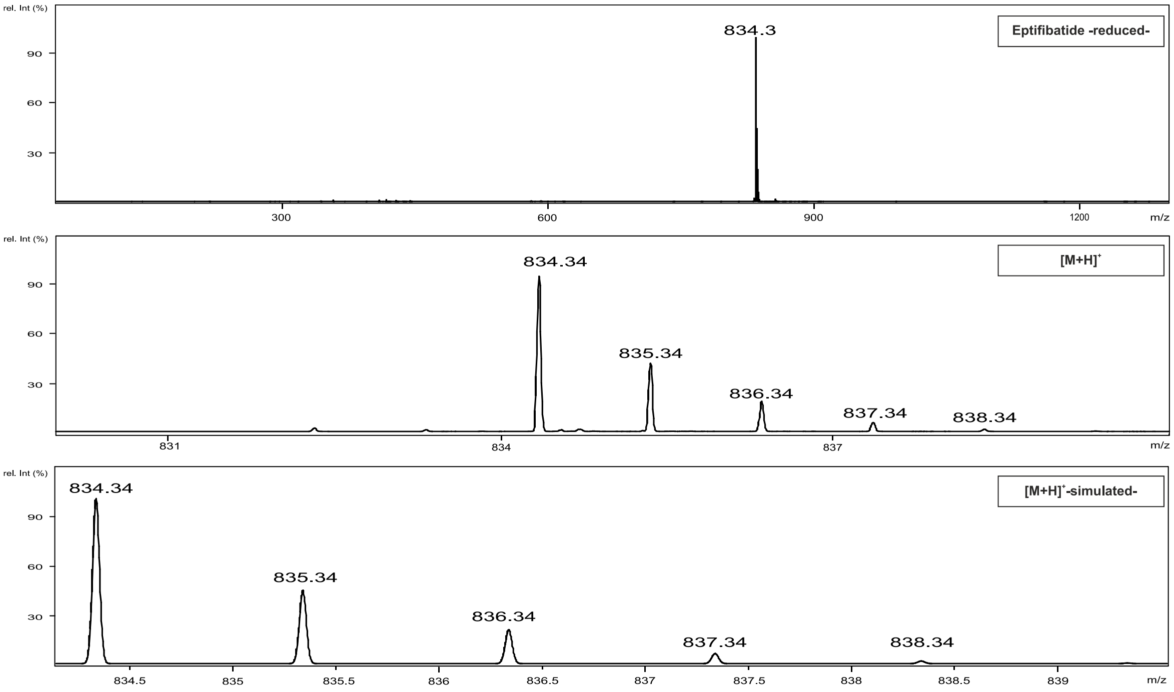

Figure 11: ESI-MS spectrum of reduced eptifibatide **2** with zoom into the area of $[M+1H]^+$ and the simulation for $[M+1H]^+$.

2.2 Spin labeled eptifibatide 3

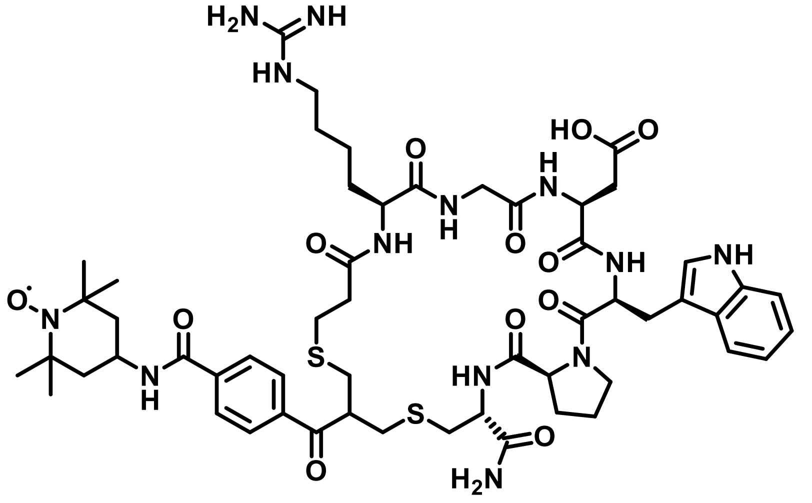

3

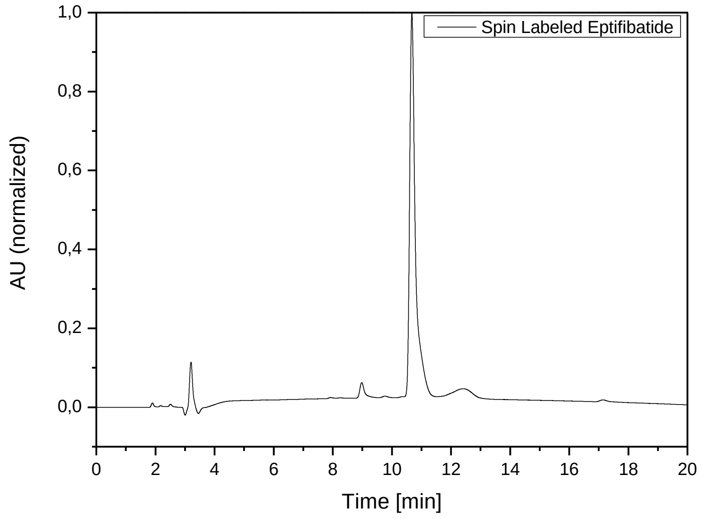

Figure 12: HPLC chromatogram of labeled eptifibatide **3** detected at 214 nm, with gradient of acetonitril in water from 20% to 80% with 0.1% TFA for 20 minutes.

- confidential -

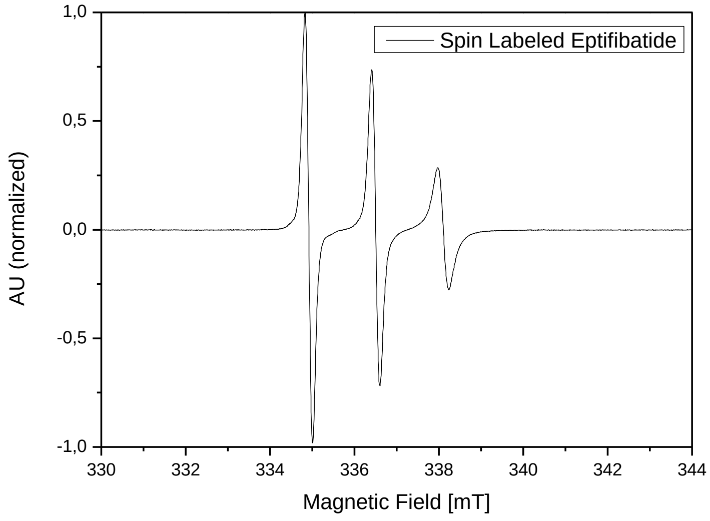

Figure 13: ESR spectrum of ca. 15 mM spin labeled eptifibatide **3**.at 20 °C.

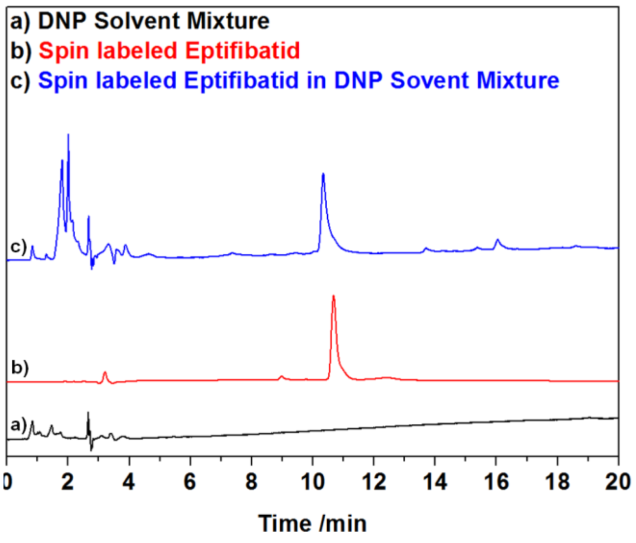

Figure 14: HPLC traces (214 nm, with gradient of acetonitril in water from 20% to 80% with 0.1% TFA for 20 minutes) illustrating the DNP solvent mixture (a), spin labeled eptifibatide **3** (b) and spin labeled eptifibatide **3** in DNP solvent mixture (c).

- confidential -
